# Use of *in vivo*-induced antigen technology (IVIAT) for the identification of *Streptococcus suis *serotype 2 *in vivo*-induced bacterial protein antigens

**DOI:** 10.1186/1471-2180-9-201

**Published:** 2009-09-18

**Authors:** Hongwei Gu, Haodan Zhu, Chengping Lu

**Affiliations:** 1 Key Lab Animal Disease Diagnostic & Immunology, Ministry of Agriculture, Nanjing Agricultural University, Nanjing 210095, PR China

## Abstract

**Background:**

*Streptococcus suis *serotype 2 (SS2) is a zoonotic agent that causes death and disease in both humans and swine. A better understanding of SS2-host molecular interactions is crucial for understanding SS2 pathogenesis and immunology. Conventional genetic and biochemical approaches used to study SS2 virulence factors are unable to take into account the complex and dynamic environmental stimuli associated with the infection process. In this study, *in vivo*-induced antigen technology (IVIAT), an immunoscreening technique, was used to identify the immunogenic bacterial proteins that are induced or upregulated *in vivo *during SS2 infection.

**Results:**

Convalescent-phase sera from pigs infected with SS2 were pooled, adsorbed against *in vitro *antigens, and used to screen SS2 genomic expression libraries. Upon analysis of the identified proteins, we were able to assign a putative function to 40 of the 48 proteins. These included proteins implicated in cell envelope structure, regulation, molecule synthesis, substance and energy metabolism, transport, translation, and those with unknown functions. The *in vivo*-induced changes in the expression of 10 of these 40 genes were measured using real-time reverse transcription (RT)-PCR, revealing that the expression of 6 of the 10 genes was upregulated in the *in vivo *condition. The strain distribution of these 10 genes was analyzed by PCR, and they were found in the most virulent SS2 strains. In addition, protein sequence alignments of the newly identified proteins demonstrate that three are putative virulence-associated proteins.

**Conclusion:**

Collectively, our results suggest that these *in vivo*-induced or upregulated genes may contribute to SS2 disease development. We hypothesize that the identification of factors specifically induced or upregulated during SS2 infection will aid in our understanding of SS2 pathogenesis and may contribute to the control SS2 outbreaks. In addition, the proteins identified using IVIAT may be useful potential vaccine candidates or virulence markers.

## Background

*Streptococcus suis *(*S. suis*) infections have been considered a major problem in the swine industry worldwide, particularly over the past 20 years. *S. suis *is a gram-positive, facultatively anaerobic coccus, and 35 serotypes (1-34 and 1/2) have been described based on their capsular antigens. Among these, serotype 2 (SS2) is the causative agent of many different syndromes worldwide, including meningitis, septicemia, arthritis, and pneumonia in humans, swine, and other animals [1]. In addition, SS2 is widely recognized as an important zoonotic agent that afflicts people in close contact with infected pigs or pork-derived products [2,3]. Two recent large-scale outbreaks of human streptococcal toxic shock syndrome (STSS) caused by SS2 in China in 1998 and in 2005 have increased public health concerns worldwide. Notably, a major outbreak of SS2 infection emerged in the summer of 2005 in Sichuan Province, China. A total of 215 cases of human *S. suis *infection were reported, and the outbreak resulted in 38 deaths and massive economic losses [4,5].

Little is known about the virulence factors of SS2. To date, only a few SS2 virulence associated factors have been identified and characterized; these include the capsular polysaccharide (CPS) [1], suilysin (SLY) [6], muramidase-released protein (MRP) [7], extracellular protein factor (EF) [8], adhesin [9], cell wall-associated and extracellular proteins [10], fibronectin- and fibrinogen-binding protein (FBP) [11], a serum opacity factor [12], and the arginine deiminase system [13,14].

An understanding of SS2-host molecular interactions is crucial for understanding SS2 pathogenesis and immunology. Conventional genetic and biochemical approaches used to study SS2 virulence factors are unable to take into account in the complex and dynamic environmental stimuli associated with the infection process. Recently, several technologies, including *in vivo *expression technology (IVET), differential fluorescence induction (DFI), signature-tagged mutagenesis (STM), transcriptional and proteomic profiling, and *in vivo*-induced antigen technology (IVIAT) have been developed to identify the pathogen genes expressed during the infection process [15,16].

IVIAT is a method that allows for the direct identification of microbial proteins expressed at sufficient levels during host infection to be immunogenic. A schematic of the IVIAT procedure was described by Rollins et al [16]. The advantage of IVIAT is that it enables the identification of antigens expressed specifically during infection, but not during growth in standard laboratory media. It was speculated that the genes and gene pathways identified by IVIAT may play a role in virulence or pathogenesis during bacterial infection [17,18]. IVIAT has been successfully used to identify arrays of *in vivo *induced proteins in *Salmonella enterica *serovar Typhi [19], *Escherichia coli *O157 [18], Group A *Streptococcus *(GAS) [17], *Vibrio cholerae *[20], and others, and these proteins have been shown to contribute to the pathogenesis or virulence of the infecting organisms. When IVIAT was applied to *E. coli *O157, it identified 223 O157 proteins expressed during human infection. Among these, four proteins--intimin-γ (an adhesin), QseA (a quorum-sensing transcriptional regulator), TagA (a lipoprotein), and MsbB2 (an acyltransferase)--had been previously identified as virulence-related proteins [18].

To identify SS2 proteins that are immunogenic and expressed uniquely during SS2 infection, we applied the newly developed and modified IVIAT method. Briefly, we screened a library of SS2 proteins expressed in *E. coli *to identify clones that were immunoreactive with convalescent-phase sera, which had been previously fully adsorbed against *in vitro*-grown SS2 and *E. coli *organisms. The adsorption process eliminates antibodies reactive with *in vitro*-expressed antigens and allows for the identification of clones expressing protein antigens that are upregulated during swine infection. Specifically, we hypothesized that by using IVIAT, we could identify proteins that play a role in the SS2-specific host-bacterium interactions unique to SS2 infection in pigs. In this study, we identified 48 putative *in vivo*-induced (IVI) proteins, which included proteins associated with bacterial cell wall structure, metabolism, regulation, molecule synthesis, substance transport and others. Of these, 10 genes were selected for analysis by real-time PCR to confirm their *in vivo *upregulation. Six genes were shown to be upregulated *in vivo*. These results suggest that these newly identified genes may contribute to SS2 pathogenesis.

## Results

### Sera selection and adsorption

IVIAT depends on the presence of antibodies directed against pathogen antigens expressed *in vivo*, so the selection of convalescent sera for use in IVIAT must be carefully considered. In this study, sera were selected that had an antibody titer of at least 10,000. All eight convalescent-phase sera, which were collected from recovered pigs as described in the materials and methods, had antibody titers above 12,800. These eight pooled convalescent-phase sera were mixed at equal volumes to create a sera cocktail for IVIAT, in order to best balance individual immune variability with the effects of dilution.

**Figure 1:**
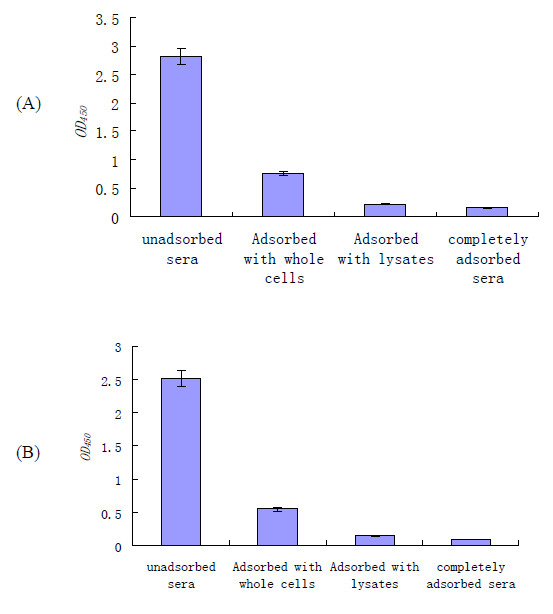
**Enzyme immunoassay reactivities of sera with lysates of an *in vitro*-grown SS2 strain after each step in sequential adsorption**. Optical density values (OD_450_) were corrected for background and for dilution during adsorption. Swine convalescent sera cocktail sets were sequentially adsorbed with SS2 whole cells, cell lysates, and *E. coli *whole cells and cell lysates. Following sufficient adsorption with all these antigens, sera were considered to have been completely adsorbed. (A) ELISA plates coated with whole SS2 cells. (B) ELISA plates coated with SS2 cell lysates. The results are expressed as means of absorbance values, and error bars represent the standard errors of the means.

The adsorption efficiency was determined by examining the immunoreactivity of the serum aliquots from the pooled swine convalescent-phase sera after each adsorption step with whole cells and cell lysates of *in vitro*-grown ZY05719. As shown in Figure 1, the immunoreactivity of the pooled sera with *in vitro*-grown SS2 progressively decreased with each round of adsorption; the decrease in immunoreactivity was particularly noticeable after the first adsorption step.

**Figure 2:**
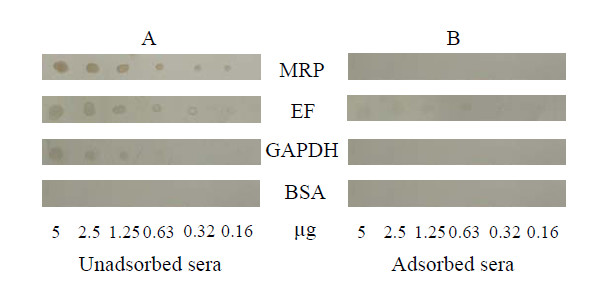
**Dot-ELISA results of reactivities of pooled unadsorbed (A) and adsorbed (B) swine convalescent sera against the three previously reported SS2 virulence-associated proteins MRP, EF, and GAPDH**. BSA was used as a negative control.

The immunoreactivity of the adsorbed pooled convalescent sera against *in vitro*-derived SS2 proteins was further assessed with dot-ELISA using the individually purified proteins MRP, EF, and GAPDH, which are reportedly expressed on the cell surface (Figure 2). Dot-ELISA results showed that unadsorbed sera strongly reacted with MRP, EF, and GAPDH (Figure 2A). However, when the sera had been completely adsorbed with *in vitro *antigens, there were no spots on the NC membrane (Figure 2B). These results further validated that antibodies specific to *in vitro *antigens had been fully removed from the convalescent-phase sera set.

### Use of adsorbed convalescent-phase sera to probe a genomic DNA expression library of the SS2 isolate ZY05719

To provide tenfold coverage of a SS2 genome (2 × 10^6 ^bp), a plasmid library containing inserts whose average size is 2 kb would contain about 5.7 × 10^4 ^independent recombinants. The SS2 genomic library, prepared from strain ZY05719 isolated from a Sichuan SS2 outbreak (Table 1), in *E. coli *DH5α consisted of approximately 6 × 10^4 ^clones for each expression vector (pET30 a, b, and c). These three libraries were used for IVIAT selection with the adsorbed convalescent sera. During the primary screening, 300 of the most intensely immunoreactive clones were selected. Following rigorous selection, 60 clones that continuously showed a strong positive reaction with the adsorbed convalescent-phase sera antibodies were identified. Their immunoreactivity was confirmed by an additional screening, in which these clones were compared with clones bearing the vectors alone without any inserts present. The positive clones were picked out and then cultured in broth. The presence of a cloned DNA insert in all 60 clones was confirmed by PCR analysis and sequencing.

**Table 1:** Bacterial strains and plasmids used in this study

Strains or plasmids	Serotype, Genotype and/or phenotype	Reference/source
Strains		
HA9801	serotype 2;*cps2J+, mrp+, ef+, sly+, gapdh+, gdh+, orf2+*	Jiangsu outbreak SS2 isolate, 1998, China
ZY05719	serotype 2; *cps2J+, mrp+, ef+, sly+, gapdh+, gdh+, orf2+*	Sichuan outbreak SS2 isolate, 2005, China
T15	serotype 2; *cps2J+, mrp-, ef-*	The Netherlands
Plasmids		
pET30(abc)	Expression vectors allowing cloning of fragments in each of three reading frames; Kan^r^	Novagen
pMRP	pET30(a) with partial *mrp *gene amplified from strain ZY05719, and cloned into *EcoRI *and *XhoI *sites, in vector, Kan^r^	This work
pEF	pET30(a) with partial *ef *gene amplified from strain ZY05719, and cloned into *EcoRI *and *XhoI *sites, in vector, Kan^r^	This work
pGAPDH	pET32(a) with partial *gapdh *gene amplified from strain ZY05719, and cloned into *BamHI *and *SalI *sites, in vector, Amp^r^	Previous work

Kan^r^, kanamycin-resistant; Amp^r^, ampicillin-resistant.

### Categorization of the IVI proteins according to the actual or putative functions of the genes identified by IVIAT

The sequencing results showed that most of the immunoreactive clones contained only a portion of the coding sequence of the relevant protein, and that these 60 clones encoded 48 different proteins. The difference in the number of positive clones and proteins is due to several clones encoding the same protein. For instance, clones 6, 34, and 73 encoded the protein *ysirk*1. The nucleotide/protein sequence data of the 48 *in vivo*-induced (IVI) proteins were deposited in the NCBI GenBank database http://www.ncbi.nlm.nih.gov, and the accession numbers that were assigned to these sequences are listed in Table 2.

**Table 2:** Proteins encoded by IVIAT selected clones

Category	Gene name	**Function and/or feature**^†^	**Bacterial cell sub-localization**^‡^	GenBank Accession number
Cell envelope structure	*ysirk1*	YSIRK Gram-positive signal peptide	cell wall	EF127690
	*ysirk2*	YSIRK Gram-positive signal peptide	cell wall	EF127720
	*ss-1616*	hypothetical protein SsuiDRAFT 0718	cell wall	EF127691
	*cwh*	Cell wall hydrolase/autolysin, peptidoglycan catabolism	cell wall	EF127695
	*trag*	TRAG protein, essential for DNA transfer in bacterial conjugation	Membrane	EF127701
	*srt*	sortase-like protein/Sortase family	Cytoplasmic	EF127708
	*nlpa*	NLPA lipoprotein	Cytoplasmic	EF127718
Regulation	*lac*	LacI:Periplasmic binding protein/LacI transcriptional Regulator		EF127711
	*hprk*	HPr(Ser) kinase, two-component signal transduction system/two-component sensor activity/regulates carbohydrate	Cytoplasmic	EF127733
	*flps*	FlpS, regulation in arginine deiminase system	Cytoplasmic	EF127749
Molecule synthesis	*ss-896*	Tagatose-6-phosphate kinase/pfkB family carbohydrate kinase	Cytoplasmic	EF127693
	*ss-1611*	Aspartate kinase/Amino acid kinase family	Cytoplasmic	EF127726
	*ss-1759*	Amino acid biosynthesis/glutamate-cysteine ligase activity	Cytoplasmic	
	*pol1*	DNA polymerase I	Cytoplasmic	EF127735
	*cpn60*	60 kDa chaperonin (Protein Cpn60) (groEL protein)/TCP-1/cpn60 chaperonin family	Cytoplasmic	EF127737
	*prpn*	Primosomal protein n	Cytoplasmic	EF127740
	*pol3*	DNA polymerase III, epsilon subunit:DNA polymerase III, alpha subunit, Gram-positive type	Cytoplasmic	EF127741 EF127751
Substance and energy metabolism	*gh3*	Glycoside hydrolase, family 3, N-terminal/Tim barrel glycosyl hydrolase superfamily. carbohydrate metabolism/hydrolase activity, hydrolyzing O-glycosyl compounds	Cytoplasmic	EF127713
	*ss-999*	hypothetical protein, carbohydrate metabolism/hydrolase activity	Cytoplasmic	EF127746
	*ss-1647*	tRNA (guanine-N(7)-)-methyltransferase/Methyltransferase superfamily, Central intermediary metabolism	unknown	EF127722
	*ss-862*	Uracil DNA glycosylase/Uracil DNA glycosylase superfamily	Cytoplasmic	EF127729
	*ss-802*	ATPase	Cytoplasmic Membrane	EF127747
	*ss-1766*	protein metabolism/ATP-dependent Clp protease, ATP-binding subunit	Cytoplasmic	EF127719
	*ss-607*	ABC transporter	Cytoplasmic Membrane	EF127697
	*sdh*	L-serine dehydratase, alpha subunit	Cytoplasmic Membrane	EF127704
	*ss-1617*	Fructokinase/Actin-like ATPase Superfamily	unknown	EF127707
	*ppc*	Phosphoenolpyruvate carboxylase		EF127727
	*ss-1305*	ABC transporter	Cytoplasmic Membrane	EF127736
Transport	*ss-1955*	conserved hypothetical protein, transport and binding	unknown	EF127692
	*ss-273*	ABC transporter, transport and binding/coupled to transmembrane movement of substances	Cytoplasmic Membrane	EF127694
	*ss-1298*	ABC transporter	Cytoplasmic Membrane	EF127714
	*ss-1829*	peptidase, S54 (rhomboid) family protein/Rhomboid family	Cytoplasmic Membrane	EF127742
	*ss-403*	PTS system sorbose subfamily IIB component	cytoplasmic	EF127754
Translation	*exc-b*	Excinuclease ABC, B subunit	Cytoplasmic	EF127698 EF127700
	*ss-887*	O-acetylhomoserine sulfhydrylase/PLP dependent aminotransferase superfamily	Cytoplasmic	EF127706
	*ss-349*	possible product: hypothetical protein SMU_684	Cytoplasmic	EF127738
	*ss-1935*	Ribosomal protein S4, bacterial and organelle form	Cytoplasmic	EF127739
Others	*ss-154*	3-phosphoglycerate kinase, phosphoglycerate kinase activity/glycolysis	Cytoplasmic	EF127702
	*ss-485*	Metal-dependent phosphohydrolase, HD subdomain/HD/PDEase superfamily	Cytoplasmic	EF127730
	*ss-1478*	Inorganic diphosphatase,	Cytoplasmic	EF127734
	*smc*	SMC protein, N-terminal: Structural maintenance of chromosome protein SMC, C-terminal: SMCs flexible hinge	Cytoplasmic	EF127743
Function unknown	g19	unknown		EF127696
	g59	Protein of unknown function DUF150	Cytoplasmic	EF127717
	g77	unknown		EF127724
	g78	unknown		EF127725
	g98	hypothetical protein Franean1DRAFT_0529		EF127732
	g130	unknown		EF127744
	g132	unknown		EF127745

† Putative functions of hypothetical proteins were determined from NCBI database and CBS Prediction Servers when available.

‡ Predicted by the PSORTb v.2.0 program.

By evaluating previously published studies of *Streptococcus *species and performing sequence alignments, we were able to assign a functional classification to 40 of the 48 proteins identified by IVIAT. Since the SS2 genomes have not been completely annotated, the genes identified by IVIAT were named after their homolog in other bacteria, when possible. When an IVI gene was unnamed, it was assigned an annotation number http://www.sanger.ac.uk/Projects/Microbes. These unnamed genes did not demonstrate good homology with the SS2 P1/7 genome determined by the Sanger Institute http://www.sanger.ac.uk/Projects/Microbes. Therefore, these genes were annotated http://www.ncbi.nlm.nih.gov. Table 2 shows the 48 IVI proteins encoded by these 60 clones that were categorized according to the actual or putative functions of the open reading frames (ORFs) identified by IVIAT. The IVI proteins were grouped into eight categories: cell envelope, regulatory, molecule synthesis, substance and energy metabolism, transport, translation, others, and those with unknown functions.

Among these 48 newly identified IVI proteins, we found three proteins previously reported to have putative roles in *Streptococcus *virulence. (i) Protein YSIRK1, when performing protein-protein BLAST with the newly identified IVI proteins against the NCBI database, we found that the protein encoded by *ysirk*1 is a potential virulence protein. Its sequence indicates that it is a subtilisin-like serine protease homologous to surface-associated subtilisin-like serine protease PrtA of *Streptococcus pneumoniae*. Interestingly, PrtA was also identified by screening a genomic expression library from *Streptococcus pneumoniae *using a convalescent-phase serum. Bethe and colleagues reported that PrtA is a highly conserved virulence factor of *Streptococcus pneumoniae*, and might be a promising candidate for a protein-based vaccine [21]. (ii) Autolysin, the autolysin encoded by *cwh *is also a reported virulence-associated factor in SS2 [22]. Most bacteria possess several autolysins that are able to degrade their cell walls, and are implicated in various biological functions including cell separation, cell wall turnover, restructuring of cell walls, and bacterial autolysis. In addition, certain autolysins have also been reported to contribute to the pathogenicity of gram-positive bacteria. For example, an intact autolytic function is required for the full virulence of *Streptococcus pneumoniae *[23]. (iii) protein TRAG, TRAG is a component of the type IV secretion system (T4SS), a virulence-associated pathway of SS2 [22]. The bacterial T4SS, which is widely distributed among the gram-negative and -positive bacteria and is ancestrally related to bacterial conjugation machines (which mediate protein and gene transfer), contributes to pathogenicity [24].

### Analysis of the *in vivo *gene expression profiles

**Figure 3:**
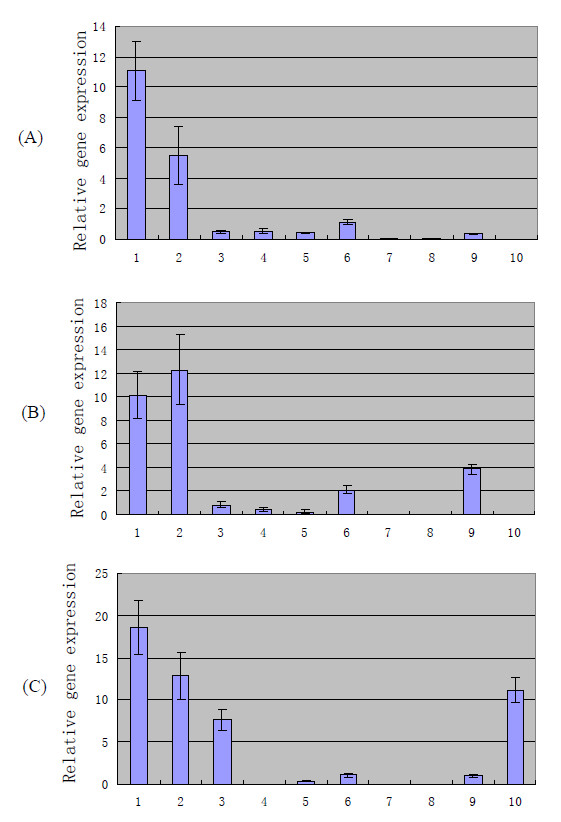
***In vivo *gene expression at 12 h (A), 24 h (B), and 36 h (C) relative to the highest level of expression *in vitro *by real-time PCR analysis**. Total bacterial RNA extracted from strain ZY05719 grown in LB broth media was used as the template to assay the *in vitro *expression levels of the 10 newly identified genes. SPF minipigs were employed as model to study the *in vivo *expression levels. Pigs were inoculated intravenously with strain ZY05719, and bacterial cells recovered from blood at 12 h, 24 h, and 36 h post-inoculation were considered as *in vivo *growth bacteria. Total bacterial RNAs extracted from in *vivo *growth bacterial cells were further analyzed by real-time PCR. To determine whether RNA expression level is induced or upregulated under *in vivo *conditions, we compared *in vivo *gene expression with the highest level of expression *in vitro*. The standard deviations are presented from three pigs each, blood collected at 12, 24 and 48 h. 1, *ss-1616*; 2, *trag*; 3, *nlpa*; 4, *srt*; 5, *cwh*; 6, *hprk*; 7, *ysirk*; 8, *ss-1955*; 9, *sdh*; 10, *ss-1298*; *gapdh *was used as reference gene.

Strain ZY05719 was selected for real-time PCR analysis because it is one of the strains isolated from the 2005 SS2 outbreak in China; ZY05719 was also used for constructing the genomic library. Of the 48 putative IVI genes, 10 (*ss-1616*, *trag*, *nlpa*, *srt*, *cwh*, *hprk*, *ysirk*, *ss-1955*, *sdh*, *ss-1298*) were selected for further analysis of gene expression by real-time PCR. We selected these genes based on their putative functions, such as involvement in cell structure, metabolism, regulation, and transport, in order to maximize the variety of genes chosen for further analysis. The *in vitro *expression of these 10 putative IVI genes was observed in early lag phase, log phase, late log phase, and stationary phase of growth, with the highest level of expression occurring at late log phase (data not shown). Before comparing the expression of these 10 putative IVI genes under the *in vitro *condition, they were first tested under *in vivo *conditions (expression after challenge with bacterial cells via intravenous inoculation measured at 12, 24, and 36 h pi). All of the putative IVI genes were expressed *in vivo *under the conditions tested (data not shown). With the exception of *ysirk *and *ss-1955*, which were expressed at 12 h pi but not at 24 and 36 h pi, and *ss-1298*, which was expressed until 36 h, the remaining 7 IVI genes were expressed at 12, 24 and 36 h post-inoculation *in vivo*. The aim of this study was to identify the genes whose expressions are upregulated *in vivo*; therefore, we determined the *in vivo *gene expression relative to the highest level of expression *in vitro*. Of the 10 analyzed genes, six genes, namely, *ss-1616*, *trag*, *nlpa*, *hprk*, *sdh*, and *ss-1298*, were upregulated *in vivo *relative to the highest level of expression of the corresponding gene *in vitro *(Figure 3).

### Location of the IVI genes on the SS2 chromosome

To learn about location of the 48 IVI genes on the SS2 chromosome, we used BLAST to identify them in the *S. suis *strain P1/7 genomic sequence (genomic sequence data were generated by the *S. suis *strain P1/7 Sequencing Group at the Sanger Institute, and can be obtained from ftp://ftp.sanger.ac.uk/pub/pathogens/ss/. Thirty-eight IVI genes were located (data not shown). Four genes (*trag*, *exc-b*, *lac*, and *ppc*) did not have high homology with P1/7, but demonstrated homology with strains *S. suis *89/1591, 98HAH33, and 05ZYH33. The remaining six genes could not be located because their sequences were short and did not show high homology with any other sequence in the database.

**Figure 4:**
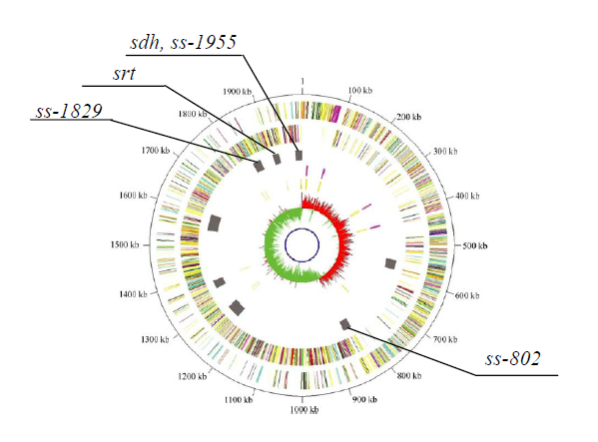
**Graphical representation of the locations of five IVI genes on the pathogenicity islands of *S. suis *serotype 2 strain P1/7**. Based on a complete analysis of the SS2 reference strain P1/7 genomic sequence, W. Wei *et al*. predicted eight putative pathogenicity islands (PAIs). When we determined the locations of the 48 IVI genes identified by IVIAT, we found five IVI genes (*sdh*, *ss-1955*, *srt*, *ss-1829*, and *ss-802*) located in four pathogenicity islands in SS2 reference strain P1/7. The genomic map was published by W. Wei *et al*., 2006 (gray bars the third ring represent eight possible pathogenicity islands).

Pathogenicity islands (PAIs) are clusters of genes that may contribute to virulence in pathogens, sometimes by responding to environmental signals [25,26]. Wei et al. (2006) predicted eight possible SS2 pathogenicity islands based on a systematic analysis of the SS2 strain P1/7 genomic sequence [27]. In this study, five IVI genes (*sdh*, *srt*, *ss-1955*, *ss-1829*, and *ss-802*) were found to be distributed in four pathogenicity islands (Figure 4) when located on the SS2 chromosome.

### Strain distribution of IVI genes

To determine whether the newly identified IVI genes are common among SS2 isolates, we examined their distribution in SS2 isolates with different backgrounds, including those isolated at different times and from different regions and sources. PCR was employed to analyze the distribution of 10 IVI genes in Chinese strains (N = 23). Twenty-three SS2 strains isolated from different regions of China in different years were analyzed, and PCR results showed that the distribution ratio of these IVI genes were as follows: *ss-1616 *(22/23, 95.7%), *trag *(23/23, 100%), *nlpa *(22/23, 95.7%), *srt *(22/23, 95.7%), *cwh *(23/23, 100%), *hprk *(23/23, 100%), *ysirk *(23/23, 100%), *ss-1955 *(23/23, 100%), *sdh *(23/23, 100%), *ss-1298 *(20/23, 87%) (details not shown). The genomic sequences of SS2 strains P1/7, 89/1591, 98HAH33, 05ZYH33 were collected from Sanger or the NCBI data library. The distribution of the 10 IVI genes in these strains was determined by nucleotide sequence alignment (Table 3). With the exception of gene *trag*, which was not found in strain P1/7, the nine remaining IVI genes were found in all four of the above strains (P1/7, 89/1591, 98HAH33, and 05ZYH33).

**Table 3:** Distributions of 10 IVI genes in SS2 strains

strain	serotype	host	region	year	Gene Name※									
					
					1	2	3	4	5	6	7	8	9	10
HA9801*	2	Pig	China	1998	+	+	+	+	+	+	+	+	+	+
ZY05719*	2	Pig	China	2005	+	+	+	+	+	+	+	+	+	+
89/1591^‡^	2	N	Canada	N	+	+	+	+	+	+	+	+	+	+
P1/7^‡^	2	N	N	N	+	+	+	+	+	-	+	+	+	+
05ZYH33^‡^	2	human	China	2005	+	+	+	+	+	+	+	+	+	+
98HAH33^‡^	2	human	China	1998	+	+	+	+	+	+	+	+	+	+

*, The distribution of the 10 IVI genes in strains was analyzed by colony PCR.

**‡, **The distribution of the 10 IVI genes in strains was performed through alignment the IVI genes with corresponding genomic sequence.

※, 1, *cwh*; 2, *hprk; *3, *ysirk; *4, *ss-1616; *5, *ss-1955; *6, *trag; *7, *sdh; *8, *srt; *9, *ss-1298; *10, *nlpa*.

N, Background not reported in related publication.

+, positive or found in the related genome sequence.

-, negative or not found in the related genome sequence.

## Discussion

*S. suis *infection is a major cause of sudden death of pigs, and is also increasingly becoming a human health concern due to its zoonotic transmission capabilities. Attempts to control the infection have been hampered by our lack of knowledge about SS2 pathogenicity. The identification and characterization of putative virulence factors and other infection-related proteins will aid in the prevention and control of SS2 disease. IVIAT provides a "snapshot" of protein expression during infection, allowing us a glimpse into the possible mechanisms by which this pathogen might counter host defenses and adapt and establish itself within the host to cause disease [18]. In the present study, we used the newly developed IVIAT method to select *in vivo*-induced proteins.

Convalescent-phase sera collected from pigs naturally infected with SS2 are ideal for IVIAT [16]. However, for multiple reasons, it is not easy to acquire convalescent-phase sera from SS2 natural infection. First, epidemiological studies have found that SS2 outbreaks are usually infrequent and only affect a small number of pigs, which can lead to underdiagnosis or misdiagnosis. Second, pigs infected with SS2 do not always show obvious clinical symptoms, and may become carriers without showing clinical signs. Finally, based on its polysaccharide capsular antigens, at least 35 serotypes of *S. suis *exist. Isolates belonging to other serotypes (such as 1, 1/2, 3, 4, 5, 7, 8 and 9) have also been associated with disease in pigs [28,29]. Common antigens had been found to be shared between SS2 and these other serotypes (unpublished data from our lab). To reduce these possible interferences, we used pigs with clear backgrounds as animal models, and convalescent sera were prepared following artificial infection.

Until recently, the exact mechanism of SS2 transmission (from pig to human or between pigs) was still poorly understood, but was thought to involve aerosol transmission or other pathways [28-30]. However, some hypotheses about the critical stages of the infection, such as bacterial invasion from the mucosal surfaces to the bloodstream, survival of the bacteria in blood, and invasion from blood into the central nervous system have been presented [28]. Regardless of the mechanism of SS2 invasion, circulation in the blood plays an important role during SS2 disease development. In addition, *S. suis *is an agent of zoonosis, afflicting people in close contact with infected pigs or pork-derived products. The organisms probably gain entry via small wounds or through inhalation [4,10,29]. Furthermore, transmission between pigs in herds through cutaneous wounds has been suggested [29]. In light of these considerations, intravenous and intramuscular inoculations were employed to assay the expression of SS2 *in vivo*, and to try to mimic natural infection (such as the middle or late stage of the infection).

In this study, we used real-time PCR to analyze the induction of the expression of IVI genes under different environmental conditions. Real-time PCR results demonstrated that the expression of six of the 10 selected genes was upregulated under *in vivo *conditions. The upregulation time points for these six genes were 12, 24, and 36 h for *ss-1616 *and *trag*, 24 h for *hprk *and *sdh*, and 36 h for *nlpa *and *ss-1298*. This upregulated expression suggests that these genes may play a significant role during the course of SS2 infection (middle, late, or whole stage of infection). The expression profiles of the other four genes (*ysirk*, *srt*, *cwh*, and *ss-1955*) showed that they were not obviously upregulated under the *in vivo *condition (Figure 3). There are two possible explanations for this result. First, since we measured the *in vivo *gene expression at 12, 24, and 36 h pi, it is possible that we missed the time when the levels of expression of these genes were high relative to the expression of the same gene *in vitro*. For instance, they may play roles in the rapid replication of SS2 during an early stage of infection. Second, the mRNA concentration might not reflect the amount of protein or antigen produced if these antigens are regulated post-transcriptionally.

The protein TRAG, which is a component of a type IV secretion system (T4SS, virulence associated pathway of SS2), was identified. The T4SS mediates horizontal gene transfer, thus contributing to genome plasticity, the evolution of infectious pathogens, and the dissemination of antibiotic resistance and other virulence traits [31]. The gene *trag *was found in 98HAH33, 05HAH33, Canada strain 89/1591, and all the tested Chinese SS2 virulent strains, but not in European strain P1/7 or the non-virulent strain T15 (data not shown). *Brucella *species require a T4SS to reach their proper niche and to replicate within host cells [32]. Whether DNA transfer through a T4SS occurs between SS2 isolates and results in an increase in virulence is unknown, and will only be answered by future studies.

Lipoproteins that are upregulated *in vivo *in other pathogenic organisms have been identified, and have been shown to be likely important for pathogenesis [33,34]. For instance, in a previous study of *Vibrio vulnificus *using IVIAT, a putative lipoprotein was also found to be induced *in vivo *when convalescent-phase sera from patients who survived *V. vulnificus *septicemia were used to screen a genomic library of this organism [35]. As for *nlpa*, almost nothing is known about the homolog of this gene in the NCBI database. The partial NLPA protein sequence was identified as a lipoprotein in the 89/1591 genome database, and shares 100% identity with a putative NLPA.

HPr kinase/P-Ser-HPr phosphatase (HprK/P) is a phosphocarrier protein of the phosphoenolpyruvate: carbohydrate phosphotransferase system (PTS). It is also a sensor of two-component signal transduction systems (TCSs) [36]. Thus, HprK/P provides a link between carbon metabolism and the development of virulence [37]. For example, the expression of several virulence genes, such as the hemolysin-encoding *hly *and the phospholipase-encoding *plcA*, is repressed when *Listeria monocytogenes *is grown on cellobiose, glucose, fructose, or other rapidly metabolizable carbon sources [38].

L-Serine dehydratase, an iron-sulfur protein [39], was identified, and this gene was also found in the Canadian strain 89/1591. During the course of the infection, alternative carbon sources (such as amino acids) are utilized by bacteria for growth due to competition for nutrients. The results of Velayudhan et al. showed that the catabolism of L-serine by serine dehydratase was crucial for the growth of *Campylobacter jejuni *under *in vivo *conditions [40]. In addition, the fermentation of amino acids produces ammonia that neutralizes the surrounding pH; this neutralization is beneficial since it protects group A Streptococcus (GAS) from acid-induced damage [41].

The protein encoded by *ss-1298 *is an ABC-type transporter, but its function is still unknown. Gene *ss-1616 *is a conserved hypothetical outer membrane protein in SS2 genome database, and almost nothing is known about this gene. It was found in all tested strains in this study, and in Canada strain 89/1591 and European strain P1/7.

Many surface proteins of pathogenic gram-positive bacteria are linked to the cell wall envelope by a sorting mechanism that recognizes an LPXTG motif, but surface proteins of *Streptococcus pneumoniae *harbor another motif, YSIRK-G/S [42]. About 20 surface proteins of *Staphylococcus aureus *carry the YSIRK-G/S motif, whereas those of *Listeria monocytogenes *and *Bacillus anthracis *do not [43,44]. While the function of the YSIRK motif has not been completely elucidated, it may contribute to the efficient secretion of a protein [43]. In the present study, four clones encoded two proteins containing this motif. Although the gene *ysirk *was only detected 12 h after SS2 infection and then disappeared, and was not strongly upregulated *in vivo*, the mature protein encoded by *ysirk1 *showed homology to the surface-associated subtilisin-like serine protease PrtA (a virulence factor) of *S. pneumoniae*[21]. However, the role of this protein during SS2 infection remains to be determined.

IVIAT enables the identification both of proteins expressed specifically during host infection but not during growth under standard laboratory conditions, and of proteins expressed at significantly higher levels *in vivo *than *in vitro*. But IVIAT has its own limitations. IVIAT will not identify all virulence-associated genes. Genes that are expressed both *in vivo *and *in vitro *and genes that are not expressed effectively in the *E. coli *host expression system will not be identified. For instance, some previously reported SS2 virulence factors, such as MRP, EF, FBPs, CPS, and SLY, could not be screened out by IVIAT in this study. We speculate that they are expressed in both *in vivo *and *in vitro *growth conditions, and therefore antibodies specific to these antigens had been eliminated during the convalescent sera adsorption steps.

Unexpectedly, some of the genes identified are likely expressed during *in vitro *growth conditions, such as DNA polymerase I and III, Primosomal protein n, protein Cpn60, and SMC protein (essential for bacterial cell division and cell wall biosynthesis). We speculate that perhaps their expression level was higher during *in vivo *growth than *in vitro *growth, and therefore they were detected by the IVIAT.

## Conclusion

Taken together, our results suggest that during the course of infection, bacterial metabolism, envelope composition, and virulence will be adjusted for bacteria to survive in the hostile environment. Bacterial pathogens sense their environment, and in response, genes are induced or repressed through spatial and temporal regulation. Genes and gene pathways identified by IVIAT may play a role in virulence or pathogenesis during host infection, and may be appropriate for inclusion in therapeutic, vaccine, or diagnostic applications. The results of this study yielded a set of potentially valuable proteins of a manageable number for future studies on SS2 pathogenicity and for the development of specific diagnostics and vaccines.

## Methods

### Bacterial strains and plasmids

The bacterial strains and plasmids used in this study are listed in Table 1. The *S. suis *strains were grown in Todd-Hewitt broth (THB) (Oxoid) or Todd-Hewitt agar (THA) (Oxoid) plates supplemented with 2% inactivated calf serum. Strain ZY05719 was originally isolated from the 2005 Sichuan SS2 infection outbreak in China. *E. coli *DH5α was used as the host strain for cloning, and *E. coli *BL21 (DE3) was used as the host strain for the recombinant proteins. The *E. coli *strains were grown in Luria-Bertani (LB) media and stored at -40°C in LB broth containing 20% glycerol. Plasmid-transformed *E. coli *cells were grown in LB medium supplemented with 30 μg/mL kanamycin (kan).

### DNA manipulation and strain construction

DNA manipulations were performed according to standard procedures [45]. All restriction enzymes, DNA polymerases, ligase, and oligonucleotide primers were purchased from TaKaRa. The *mrp, ef*, and *gapdh *genes were amplified by PCR, and each gene was separately ligated into pET expression vectors to construct 3 recombinant expression plasmids (Table 1). These recombinant expression plasmids were separately introduced into *E. coli *BL21 (DE3) and induced to overexpress recombinant proteins.

### Indirect ELISA and dot-ELISA

An indirect enzyme-linked immunosorbent assay (ELISA) was used for screening the swine sera with the *in vitro*-derived SS2 antigens. In brief, microtiter plates (Costar) were coated with SS2 antigen (whole cells and cell lysates). Following incubation and blocking, 100-μL dilutions (1:200-1:51,200, V/V) of sera were added to the wells. The subsequent ELISA protocol was performed as previously described [46]. 3,3',5,5'-tetramethylbenzidine (TMB, Amresco) was used as the substrate, and the optical density at 450 nm (OD_450_) was determined with an ELISA reader (BIO-RAD550). The antibody titer was defined as the highest serial dilution of serum for which the OD_450 _value was two standard deviations above the mean OD_450 _of the negative controls (without primary antibody).

To assay for antibodies specific to MRP, EF, and GAPDH, successively diluted nickel affinity-purified recombinant-expressed MRP, EF, and GAPDH proteins were spotted on a nitrocellulose (NC) membrane (Millipore). Dot-ELISA was performed according to the standard procedure with minor modifications [46]. The reactions were developed with 3,3'-diaminobenzidine (DAB, Amresco) solution with 0.1% H_2_O_2_.

### Swine convalescent sera and control sera

Recently, a specific pathogen-free (SPF) piglet has been developed as an animal model for studying *S. suis *[47,47]. Animal experiments were performed as previously reported with minor modifications [48]. All animal experiments were conducted in accordance with national guidelines and were approved by the office of laboratory animal, Shanghai Jiao Tong University and Shanghai administration committee of laboratory animals.

#### Convalescent sera

To prevent any contact with infectious agents, SPF Bama minipigs and healthy piglets were housed in independent units with absolute filters. Prior to challenge, all the pigs were negative for SS2-specific antibodies, as determined by an ELISA test. SPF minipigs (n = 8, Guizhou line, 7 weeks old) were randomly grouped into 2 units (4/unit, named as group 1 and 2) and piglets (n = 12, 8 weeks old) into 2 units (6/unit, named as group 3 and 4). Bacterial suspensions in THB with 10% inactivated bovine serum were prepared and adjusted to a concentration of 1 × 10^8 ^colony forming units (CFU)/mL of *S. suis*. These pigs were challenged with 2 mL of strain ZY05719 (1 × 10^8^CFU/mL), intramuscularly (i.v.) for group 1 and 3, and intravenously (i.m.) for group 2 and 4, respectively. The pigs were monitored daily post-inoculation (pi) for clinical signs, notably fever and central nervous system dysfunctions such as opisthotonos, tremors, and nystagmus. The rectal temperature was recorded daily. No inflammation was observed at the injection sites. Intramuscularly challenged pigs died naturally between 4 and 8 days after experimental infection, while intravenously challenged pigs died between 2 and 7 days. The pigs, 3 minipigs (1 for i.v. group and 2 for i.m.) and 5 piglets (2 for i.v. group and 3 for i.m.), that recovered after being challenged were used in the subsequent experiments performed in this study. The antibody titer against a homologous strain was determined by indirect ELISA every week after challenge. At week 4, the animals were sacrificed and bled. The sera were collected and kept frozen at -40°C. The flowchart of piglet infections was as shown in Additional File 1: Figure S1. Convalescent sera collected from the recovered pigs were used for IVIAT selection.

#### Positive control sera

SS2-positive sera were prepared from 3 SPF minipigs immunized with inactivated ZY05719 whole cell bacteria (2 mL of 1 × 10^8 ^CFU each) 4 times at 2-week intervals. Ten days after the last injection, the antisera were pooled and used as the positive control in ELISA tests.

#### Negative control sera

To reduce variability animal to animal, serum samples were obtained from healthy SPF minipigs prior to SS2 infection, negative in ELISA test, used as the negative control for IVIAT or ELISA.

### Adsorption of swine convalescent-phase and control sera

To compensate for variations in the immune responses of individual pigs, equal volumes of convalescent sera from 3 minipigs and 5 piglets were pooled and extensively adsorbed with *in vitro*-derived SS2 antigens to completely remove all antibodies that recognize the antigens that are expressed under the *in vitro *condition.

The adsorption protocol has been described previously [20]. Briefly, a Protease Inhibitor Cocktail Set II (Merck) formulated for bacterial cells and containing 4-(2-aminoethyl) benzenesulfonyl fluoride (AEBSF; 20 mM), EDTA (85 mM), bestatin (1.7 mM), pepstatin A (2 mM), and E-64 (0.2 mM) was prepared per the manufacturer's instructions and then added to intact cells and cell lysates at a dilution of 1:10 (V/V). The successive adsorption steps were performed six times with whole bacterial cells, three with native cell lysates, and one with heat-denatured ZY05719 cell lysates and *E. coli *BL21(DE3) that contain unmodified pET-30abc expression plasmids (Novagen), as described[15,20]. Cell lysates were prepared by sonication, and the protein concentration determined by using spectrophotometer (Smartspec™, BIO-RAD). The cell lysates were first coated onto nitrocellulose membranes and the corresponding antibodies adsorbed to remove antigen-antibody complexes. The resultant adsorbed serum was divided into aliquots that were stored at -40°C. To check the efficacy of each adsorption step, a 10-μL serum aliquot was removed after each adsorption and analyzed with ELISA against either whole SS2 cells or SS2 cell lysates.

### Construction of a genomic expression library of the SS2 strain ZY05719

An expression library was constructed with the pET-30abc series of expression vectors, which permit the cloning of inserts into each of the three reading frames under the transcriptional control of the T7 phage promoter. Each vector DNA was digested with *Bam*HI, subjected to agarose gel electrophoresis, purified by using a gel extraction kit (TaKaRa), and treated with shrimp alkaline phosphatase (TaKaRa). Genomic DNA from strain ZY05719 was extracted and partially digested with *Sau*3AI. Next, we ligated each of the three vectors separately with genomic DNA fragments to create three expression libraries. These libraries were electroporated into competent *E. coli *DH5α as described previously [18,20]. We assessed the resulting libraries by subjecting a random sample to PCR in order to determine the frequency and size of the inserts. More than 98% of transformants contained inserts of sizes ranging from 0.1 to 4 kbp.

### Screening the antigens identified by IVIAT against swine convalescent-phase sera

Immunoscreening was performed according to the procedure described by Sambrook et al. [45]. In short, an aliquot of the library with *E. coli *BL21 (DE3) as the expression host was diluted and spread on sterile NC membranes that were overlaid on kan/LB plates. After overnight incubation at 37°C, the colonies were lifted onto new sterile NC membranes, and after a 5-h incubation at 37°C, these membranes with the lifted colonies (colony side up) were overlaid on fresh kan/LB plates containing 1 mM isopropyl-D-thiogalactopyranoside (IPTG, Amresco) and incubated overnight at 37°C to induce gene expression of the cloned inserts. The plates were exposed to chloroform vapors for 15 min for partial bacterial lysis and for the release of the induced proteins. Each membrane was removed, blocked with 5% skim milk, and reacted with a 1:100 (V/V) dilution of the pooled adsorbed convalescent-phase sera at room temperature. The clones that reacted with the antibodies in the adsorbed sera were detected by using peroxidase-conjugated staphylococcal protein A (SPA) and visualized with an Enhanced chemiluminescence (ECL) kit (Pierce). The immunoreactive clones were identified by their position on the master membrane. Each positive clone was purified at least two additional times and confirmed as immunoreactive to the adsorbed sera [18,20]. Plasmids from individual positive reactive clones were purified, and the DNA inserts were sequenced in both directions by using pET30-specific primers.

### Bioinformatic analysis

Analysis of sequence homologies, protein families, and conserved domains was performed using NCBI BLAST http://blast.ncbi.nlm.nih.gov, information from the Sanger Genome Centre http://www.sanger.ac.uk/Projects/S_suis, and PFAM http://pfam.sanger.ac.uk. The putative functions of the newly discovered proteins were assigned using the CBS Prediction Servers http://www.cbs.dtu.dk/services/ProtFun. The cellular localizations of these proteins were predicted using PSORTb v2.0 http://www.psort.org/psortb/.

### Real-time PCR analysis

Gene expression was tested by subjecting the RNA of the bacteria grown under standard laboratory conditions to real-time PCR, and the results were compared to those obtained for bacteria recovered from infected pigs.

#### *In vitro *culture

Duplicate cultures of ZY05719 grown under *in vitro *conditions were harvested at OD_600_s of 0.1, 0.2, 0.4, 0.6, and 0.8. OD_600_s in the ranges of 0.1-0.2, 0.2-0.6, and 0.6-0.8 correspond to the lag phase, log phase, and stationary phase, respectively. The bacterial pellet was snap frozen in liquid nitrogen and stored at -80°C.

#### *In vivo *gene expression

Three SPF Bama minipigs were inoculated intravenously with ZY05719 for analyzing gene expression under *in vivo *conditions. The bacterial cells were separated from blood by centrifuging at different speeds. Blood samples were pooled at 12, 24, and 36 h pi, centrifuged at 2,000 rpm to remove blood cells, and repelleted at 12,000 rpm to collect bacterial cell pellets that were subsequently snap frozen in liquid nitrogen and stored at -80°C.

#### Real-time PCR

Bacterial total RNA was extracted using RNAprep Bacteria Kit (TIANGEN, China), and residual genomic DNA was removed by using a QIAGEN RNase-Free DNase Set (Qiagen) according to the manufacturer's instructions. DNase-treated RNA samples were reverse transcribed by using a first-strand cDNA synthesis kit (TaKaRa) according to the manufacturer's recommendations. The controls for cDNA synthesis and DNase treatment included two negative controls: one with no RNA template and one without reverse transcriptase.

Quantitative real-time PCR (qPCR) assays were performed by using a Chromo4 system (BIO-RAD) and a SYBR-Green PCR kit (Takara). All qPCR reactions were performed in a final volume of 25 μL containing 12.5 μL Premix Ex Taq mix (2×), 0.5 μL (10 μM) of each primer, 2 μL cDNA, and 9.5 μL double-distilled water (ddH_2_O). The protocol followed for each qPCR was as follows: hot start at 95°C for 10 s, followed by 45 cycles at 95°C for 5 s, 60°C for 20 s. Data were collected and analyzed using Opticon Monitor software V3.1 (BIO-RAD). To normalize the data, primer pairs were designed to amplify the gene glyceraldehyde-3-phosphate dehydrogenase (*gapdh*) as housekeeping control. Based on the gene classification, 10 genes were selected for the PCR amplification and the specific primer sets that were used are listed in Table 4. The specificity of each resulting amplicon was validated with its corresponding melting curve. The relative level of expression was calculated by comparing the difference in the threshold cycle number of the gene of interest gene with that of the reference gene.

**Table 4:** Primers used for real-time PCR in this study

gene	Sequences of primers (5' to 3')	Amplicon size (bp)
*cwh*	TGGTAAATGCCCCATCTAGTC	137
	GGCTGTAACACCAATAATTTCC	
*hprk*	GAAACCCCTGTTGTCATAGTGG	126
	CAATTCTCCCGATAGACGACTG	
*ss-1616*	ACAGGGAATAAGCATCAGCG	119
	ATGTAGTTACGCTCCGCCTT	
*ysirk*	GCACTTTTATTGCCACGGATT	160
	CAGCACCTTGTTGTCTCGGA	
*gapdh*	TTGGAAGCTACAGGTTTCTTTG	98
	TTACCACCAGGAGCAGTGACA	
*ss-1955*	ATCAGGTTCTAACATTGTTGCG	122
	TAACGCCCCCCTCTAACAAG	
*srt*	GGTCGACGAAGTGTCATTGC	123
	ATACGTCAGCGTCCTCCCAC	
*nlpa*	CTGCAACCTGGTCACCAAATAC	129
	ACCCCGGAAAAGTTACGTATGA	
*sdh*	TAGAAGTCCCTTGTGTCAGACG	134
	AGATCCCACTTGGTACATAGCG	
*ss-1298*	TGGATATCGACAGCAAGGAG	156
	CATAGTCGCCCAAATAGAGC	
*trag*	TCGTGACTTGATGACGGCTG	167
	GATAATGCCACCAGCGTTCA	

### Colony PCR analysis

To learn about gene distribution in diverse SS2 isolates with different backgrounds, colony PCR was used. The primers used to detect the 10 IVI genes were same as the oligonucleotides for qPCR (Table 4). Single SS2 colonies were picked from THA plates, suspended in 50 μL of _dd_H_2_O and boiled for 10 min to make DNA lysates. Each was assayed using the appropriate primer sets by PCR. PCR reactions were carried out using Taq polymerase according to the manufacturer's recommendation (TaKaRa).

## Authors' contributions

HWG carried out the IVIAT selection, participated in the sequence alignment, performed real-time RT-PCR and drafted the manuscript. HDZ carried out the animal experiments and participated in the PCR amplification. CPL conceived of the study, participated in its design and coordination. and critically revised the manuscript. All authors read and approved the final manuscript.

## Supplementary Material

Additional file 1 **Swine convalescent sera preparation**. The data provided represent the preparation of swine convalescent sera. * Time-point of antibody check. ‡ Sacrificed and serum collection. † Number of recovered pigs, and antisera were used as convalescent sera for IVIAT selection. § sera were collected and used as negative control. Click here for file
