# Supplementary figures and images for: Use of in vivo-induced antigen technology (IVIAT) for the identification of Streptococcus suis serotype 2 in vivo-induced bacterial protein antigens
Source: BMC Microbiol. 2009 Sep 18;9:201. doi: 10.1186/1471-2180-9-201 (PMC2758882; doi:10.1186/1471-2180-9-201)

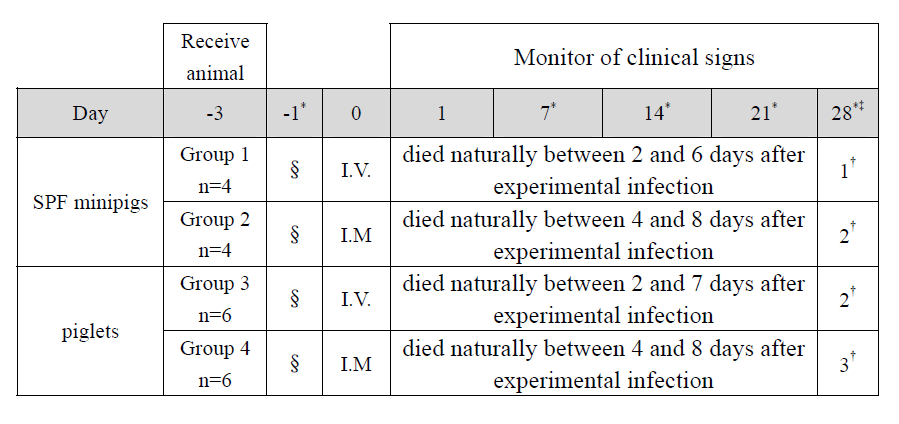

Supplement: Additional file 1 — Swine convalescent sera preparation. The data provided represent the preparation of swine convalescent sera. * Time-point of antibody check. ‡ Sacrificed and serum collection. † Number of recovered pigs, and antisera were used as convalescent sera for IVIAT selection. § sera were collected and used as negative control. [file 1471-2180-9-201-S1.TIFF]
